# A TRP Family Based Signature for Prognosis Prediction in Head and Neck Squamous Cell Carcinoma

**DOI:** 10.1155/2022/8757656

**Published:** 2022-01-31

**Authors:** Fangfang Pan, Kai Wang, Mengmeng Zheng, Yuan Ren, Wenjuan Hao, Jiangyu Yan

**Affiliations:** Department of Otorhinolaryngology Head and Neck Surgery, Hwa Mei Hospital, University of Chinese Academy of Sciences, Ningbo 315010, China

## Abstract

**Purpose:**

Head and neck squamous cell carcinoma (HNSCC) is a classical type of head and neck cancers, with heterogeneous clinical outcome. This project is set out to create a robust risk signature based on TRP family genes (TFGs) for prognosis evaluation in HNSCC.

**Methods:**

Based on the HNSCC sample data from the TCGA website, we integrated expression profile of TFGs for 490 HNSCC cases. We explore the interactions among TFGs using STRING tool. The TFGs-based signature (TFBS) was created by Cox relative analyses. In addition, we conducted GSEA to identify the underlying signaling pathways of the specific TFGs in HNSCC. The immune landscape of HNSCC patients was analyzed by CIBERSORT and ssGSEA algorithms.

**Results:**

A total of 6 TFGs (TRPC1, TRPC3, TRPC6, TRPV2, TRPV4, and TRPM8) closely associated with prognosis of HNSCC cases were screened to create TFBS. TFBS predicted that the TFBS-high group presented dismal patient outcome. Cox regression revealed the favorable independent value of TFBS. ROC analysis showed the robust power of TFBS for prognosis forecasting. GSEA determined several crucial pathways related with HNSCC, which are the p53 pathway, TNF-alpha signaling via NFKB, and hypoxia. Moreover, immune-related analysis showed that patients in the TFBS-high group were more likely in immunosuppressive status.

**Conclusion:**

Our proposed TFBS could serve as a favorable indicator to forecast the survival outcome of HNSCC cases and offer prominent therapy guidance.

## 1. Introduction

Head and neck squamous cell carcinoma (HNSCC) is a frequent neoplasm developing in the head and neck region, including tongue, mouth, neck, nasopharynx, larynx, and throat [1]. Currently, the clinical therapy of HNSCC is still based on surgery and other adjuvant treatment methods, such as systemic chemotherapy, local radiotherapy, and immunotherapy [2]. Despite the continuous improvement of diagnostic techniques and clinical therapy, the patient outcome of oral cancer has not been improved significantly, with a dismal 5-year survival rate below 50% [3]. It is hard to forecast the clinical outcome of HNSCC due to its occult heterogeneity and various etiological factors. Since the incubation period of HNSCC is long and the early clinical symptoms are not obvious, more than 60% of the patients have been diagnosed at the middle and advanced stages [4]. Therefore, it is urgent to exploit a robust and reliable signature to enhance the prediction of HNSCC prognosis.

Transient receptor potential (TRP) is a classic cation channel located on the surface of biological membrane, penetrating Ca^2+^, Mg^2+^, Na^+^, K^+^, and other cations. The TRP superfamily can be divided into 7 subfamilies: TRPA (ankyrin), TRPC (canonical), TRPM (melastatin), TRPML (mucolipin), TRPN (NOMP-C), TRPP (polycystin), and TRPV (vanilloid) [5]. The channel has 6 transmembrane structural domains in the cell membrane, exercising their functions as subunits assembled into homo or heterotetramers [6]. TRP channels are classical calcium channels that allow extracellular calcium to flow through the cell membrane into the cell, and their dysfunction is bound up with malignant behavior of tumors [7].

Accumulating evidence suggests that TRP family genes (TFGs) play a central part in regulation of malignant behavior in various tumors, including gastric cancer (GC), breast carcinoma, and epithelial ovarian carcinoma [8–12]. For example, Gao et al. found that TRPV1 in TRP channel family genes uniquely inhibits the development of GC through the Ca/CaMKK*β*/AMPK pathway. Also, a higher expression of TRPV1 is positively correlated with better prognosis of patients with GC [10]. In ovarian cancer (OC), Liu et al. revealed that TRPM7 could regulate epithelial-mesenchymal transition (EMT) by activating calcium influx [13]. As unearthed by Song et al., TRPV6 was higher in pancreatic cancer (PC) cases than in normal controls. TRPV6 knockdown could greatly block cell viability and metastasis and promote apoptosis, suggesting that it might be a favorable indicator for PC [14].

Here, we detected the relationship between TFGs expression patterns and prognosis of HNSCC and further set up a TFG-based prognostic model which can offer valuable medical potency for prognostic prediction and individualized treatment for HNSCC.

## 2. Methods

### 2.1. Patients and Datasets

By processing the data of the HNSCC in TCGA, the mRNA-seq expression profiling and clinical information of 490 HNSCC cases were collected. Next, we employed the scale method to normalize the mRNA expression profiles by limma package in *R* software. Then, a total of 28 TRP family genes (TFGs) were collected from the previous reports and studies [7, 15, 16] and are shown in Supplementary Table S1.

### 2.2. Identification of the Interaction Network

The STRING website was implemented to study the protein-protein interactions (PPIs) of 28 TFGs. In this study, a PPI score of 0.4 was set as the threshold. Cytoscape software was used to screen the hub genes with the maximum cluster centrality (MCC) algorithm and the visualize the PPI network.

### 2.3. Identification of TRP Family Based Signature

To develop a favorable TRP family based signature (TFBS), all the HNSCC samples were randomly divided into a training cohort and a validation cohort. In the training cohort, we used the univariate Cox method to determine the potential prognostic factors from 28 TFGs (*p* < 0.05). Furthermore, significant prognosis-related factors from univariate regression were analyzed by the multivariate Cox method to generate a TFBS. The risk value of TFBS = ∑exp(TFG*s*)*∗β*. The *β* is the coefficient of each prognostic TFGs calculated by Cox methods.

### 2.4. Construction of a TFBS-Based Nomogram

Cox relative regression methods that incorporated age, gender, stage, and risk score were implemented to confirm the independent power of the TFBS using survival package in *R*. Moreover, we also set up a nomogram based on TFBS to strengthen the predictive ability of TFBS. Verification of the nomogram was assessed by calibration curves.

### 2.5. Immunity Patterns of the Signature

After integrating the gene sequencing data in TCGA and standard annotation of 22 types of immunocyte, we determined the immune landscape of HNSCC patients by the CIBERSORT algorithm. *P* < 0.05 was set as the threshold. In addition, single-sample gene set enrichment analysis (ssGSEA) was applied to immune activity between two risk subgroups according to TFBS.

### 2.6. Tumor Mutation Analysis

The mutation data of the TCGA-HNSCC dataset were analyzed using the maftools. The tumor mutational burden (TMB) was generated using the following formula:

(1) $$\begin{array}{c} \text{TMB}=\frac{\text{Total mutation}}{\text{Total covered bases}}\times 10^{6}. \end{array}$$

### 2.7. Gene Set Enrichment Analysis (GSEA)

GSEA was performed to unearth the underlying tumor hallmarks and signaling pathways associated with TFBS based on the Hallmark and KEGG terms. We determined greatly enriched gene sets with *p* value <0.05 after 1000 substitutions.

### 2.8. Verification of Expression Values of TFBS

To test the expression pattern of 6 TRPs of the TFBS model, we conducted the differentiation expression analysis by limma package in *R* project.

### 2.9. Statistical Analysis

R project (3.6.3) was used for all statistical analyses. To detect the survival differences between the two risky cohorts, the Kaplan–Meier (K-M) method was applied. The reliability of the TFBS was confirmed using receiver operating characteristic (ROC) analyses. *P* < 0.05 was considered statistically significant.

## 3. Results

### 3.1. Characterization of TRP Family Genes

**Figure 1:**
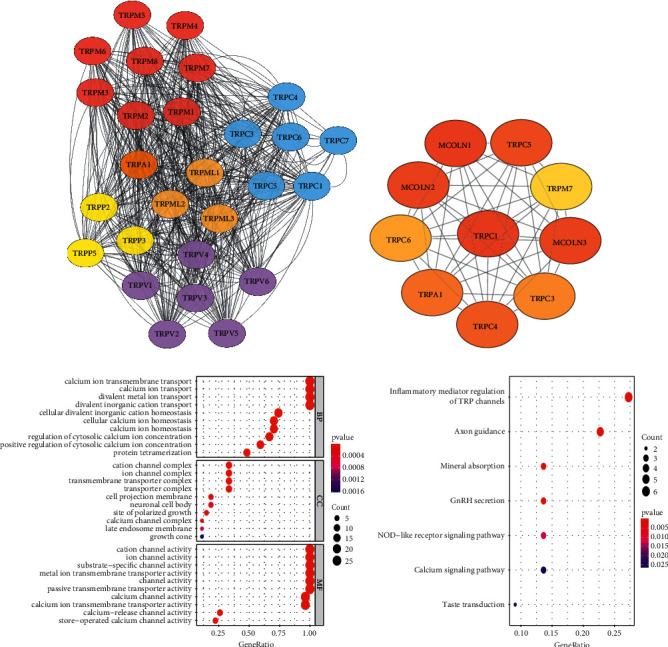
Characterization of TRP family genes (TFGs). (a) PPI network of the 28 TFGs. (b) The top 10 hug genes of the TFG-based PPI network. (c) GO functional analysis for the 28 TFGs. (d) KEGG enrichment analysis for the 28 TFGs.

To uncover the interactions among 28 TFGs, we first created a PPI network (Figure 1(a)). One of the TRP family genes (TRPC2) was not found in the STRING database. Then, we employed the MCC algorithm to screen 10 hub genes with highest interaction scores using Cytoscape, including TRPC1, TRPC3, TRPC4, TRPC5, TRPC6, TRPA1, TRPM7, TRPML1, TRPML2, and TRPML3 (Figure 1(b)). As shown in Figure 1(c), calcium ion transmembrane transport was greatly enriched for the biological process. The results of KEGG disclosed that 28 TFGs were greatly involved in inflammatory mediator regulation of TRP channels and calcium signaling pathway (Figure 1(d)).

### 3.2. Establishment and Verification of the TFBS

First, a total of 245 cases were randomly included in the training cohort and create a risk model for these 245 patients. Next, we applied univariate Cox analysis to determine 15 TFGs which were dramatically associated with prognosis of HNSCC cases (*p* < 0.05). Then, 15 prognostic TFGs were analyzed with the multivariate Cox method. Finally, we successfully developed TFBS based on 6 hub TFGs, including TRPC1, TRPC3, TRPC6, TRPV2, TRPV4, TRPM8 (Table 1). The risk factor = (TRPC1 × (0.2075)) + (TRPC3 × (0.1439)) + (TRPC6 × (0.1553)) + (TRPV2 × (0.2698)) + (TRPV4 × (0.2947)) + (TRPM8 × (0.0778)). All HNSCC patients were classified into high and low risk groups according to the cutoff value of the risk score.

**Figure 2:**
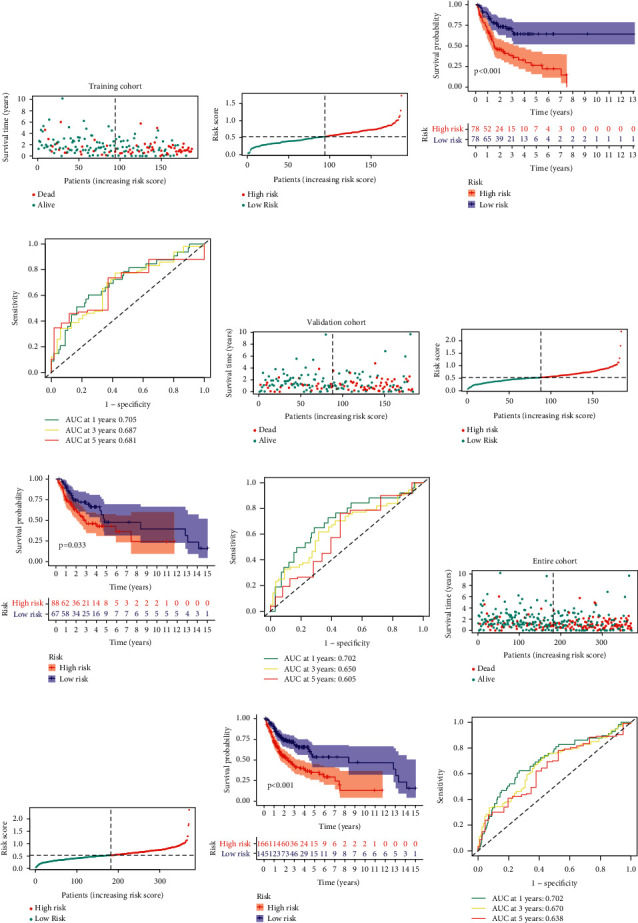
Predictive value of ARS. (a) The patient outcome of HNSCC. (b) The layout of growing risk scores. (c) Survival analysis for two groups. (d) ROC curves to confirm the performance of TFBS in the training set. (e–l) Present similar results verified in the validation and entire sets.

**Figure 3:**
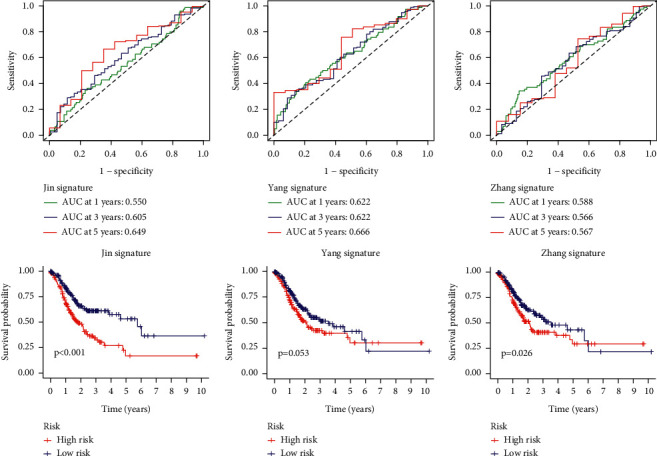
Comparison of TFBS with other established signatures. (a) ROC and KM curves of the Jin signatures. (b) ROC and KM curves of the Yang signatures. (c) ROC and KM curves of the Zhang signatures.

The performance of TFBS for forecasting clinical outcomes of patients is shown in Figure 2. In the discovery set, survival analyses suggested that TFBS-low patients had greatly favorable prognosis than TFBS-high patients (Figure 2(c)). Subsequently, ROC analysis was implemented to test the reliability of TFBS. The results showed that AUC values were 0.705, 0.687, and 0.681 for 1, 3, and 5-year survival, respectively (Figure 2(d)). Moreover, similar results were found in the validation and entire cohorts, suggesting that TFBS has robust ability for prognosis prediction (Figures 2(g) and 2(h); Figures 2(k) and 2(l)). Also, we implemented ROC analyses to compare the prediction ability of TFBS with other established risk models [17, 18], and our nominated TFBS had the highest AUC values (Figure 3), suggesting the robust ability of TFBS for prognosis prediction.

### 3.3. Validation of Six Hub Genes of TFBS

**Figure 4:**
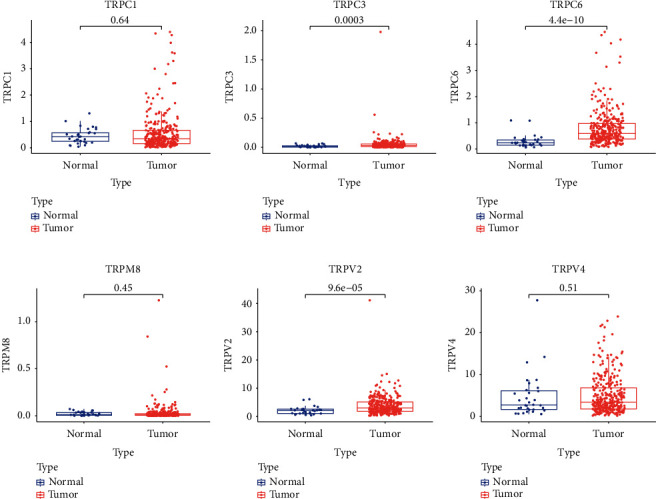
Expression levels of the 6 signature genes.

To detect the expression patterns of 6 signature genes, we performed differentiation analysis. The results disclosed that TRPC3, TRPC6, and TRPV2 were upregulated in HNSCC specimens compared with normal tissues, but TRPC1, TRPM8, and TRPV4 had no statistical differences between two groups (Figure 4).

### 3.4. Construction of a Prediction Nomogram

**Figure 5:**
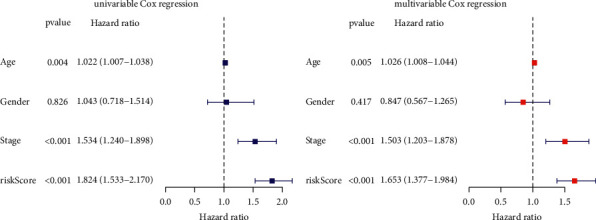
Determination of the independence of TFBS. (a) Univariate regression analysis. (b) Multivariate regression analysis.

**Figure 6:**
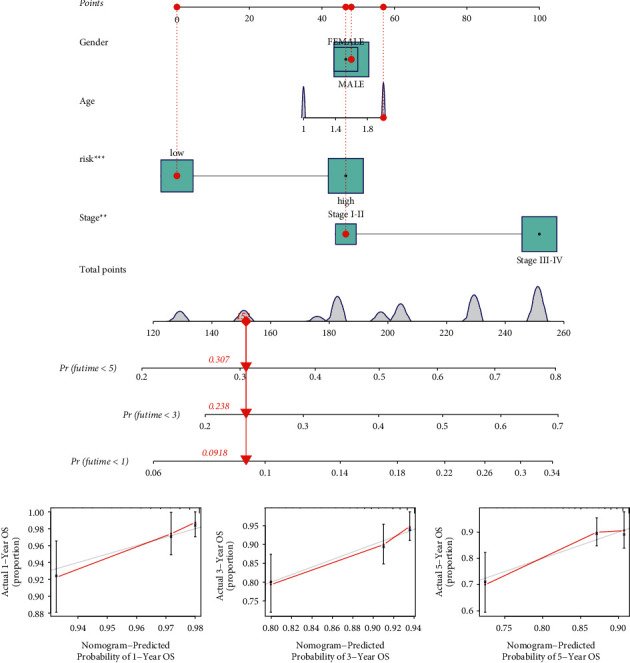
Development of the TFBS-based nomogram. (a) The nomogram for forecasting patient 1, 3, or 5-year survival. (b) The calibration plots for confirming the reliability of the nomogram.

Cox relative regression methods were implemented to test the independent power of TFBS on the basis of prognosis of HNSCC cases. As indicated by the univariate Cox method, the risk score was notably meaningful for forecasting patient outcome (Figure 5(a)). After performing the multivariate Cox method, the risk score was proved to be independent of other clinicopathological characteristics (Figure 5(b)). Furthermore, age, gender, stage, and TFBS were selected to generate a nomogram which could open up the predictive value of TFBS (Figure 6(a)). As shown in Figure 6(b), calibration curves reveal the outstanding reliability of TFBS-based nomogram.

### 3.5. Correlation between TFBS and Immune Landscape

**Figure 7:**
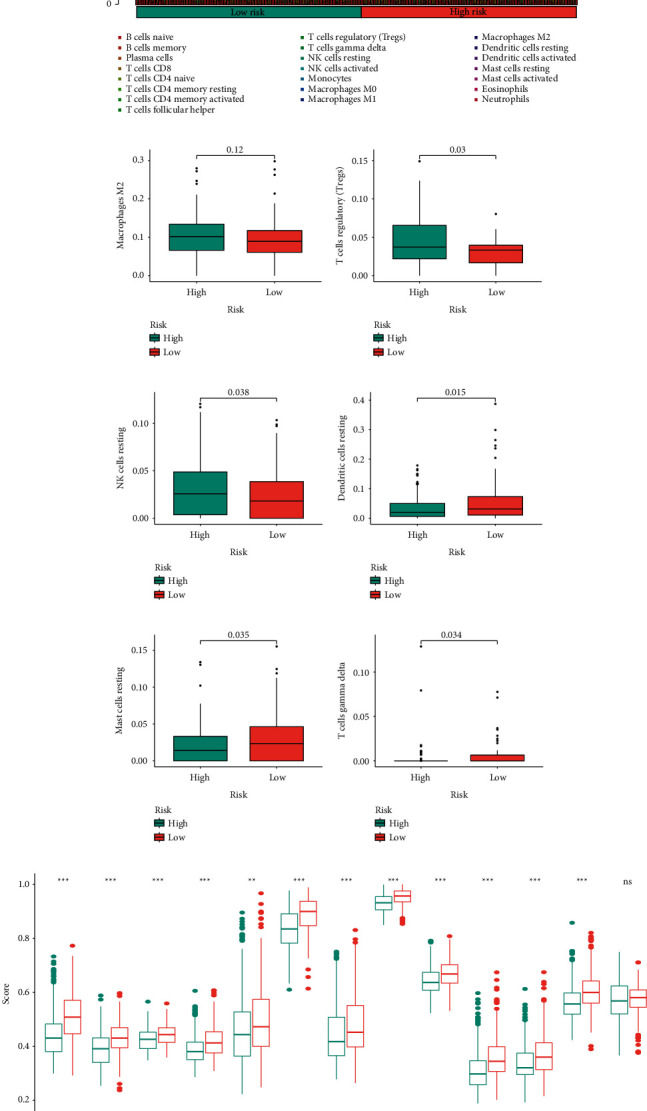
Immune analysis of TFBS. (a) Relative frequency of immunocyte infiltration in HNSCC. (b–g) Box plots showing markedly immunocytes between two groups. (h) Immune-related function analysis for two groups by the ssGSEA method.

We first estimate the differences in the immunocyte infiltration between two subgroups (Figure 7(a)). The TFBS-high group displayed remarkably higher proportions of M2 macrophage and T cells regulatory (Tregs) and resting NK cells (Figures 7(b)–7(d)), while remarkably lower proportions of resting dendritic cells, resting mast cells, and gamma delta T cells (Figures 7(e)–7(g)). In addition, we analyzed the immune-related functions in terms of HNSCC samples by ssGSEA. The results showed that most of immune-related functions were upregulated in the TFBS-high group, pointing out that these patients might be associated positively with immunosuppressed status (Figure 7(h)).

### 3.6. TMB Analysis of the TFBS

**Figure 8:**
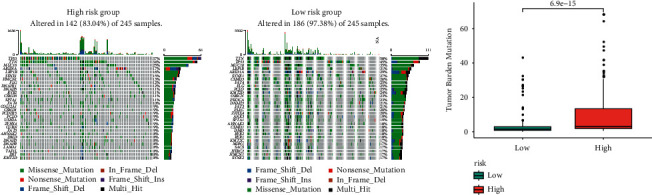
Tumor mutation analysis. (a) The hub-mutated markers in both groups. (b) TMB in both risk groups.

Given the predictive role of TMB in the immunotherapy, we further performed TMB analysis. Mutation-associated genes (MAGs) in both groups are shown in Figure 8(a). TP53, MUC16, TTN, ARID1A, and LRP1B were the top 5 MAGs. Also, we found that TMB was greatly higher in the high-TFBS group, indicating that the high-TFBS group is more likely to benefit from immunotherapy (Figure 8(b)).

### 3.7. GSEA Enrichment of the TFBS

**Figure 9:**
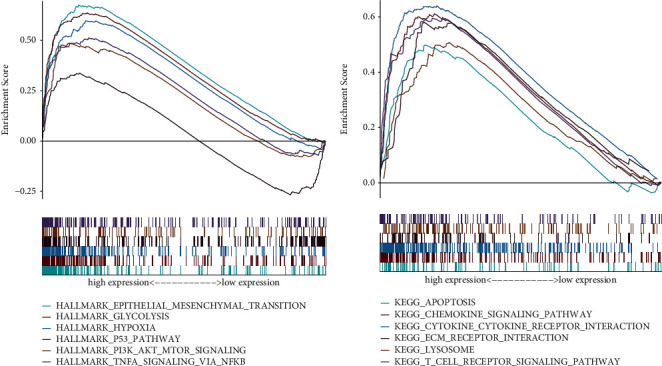
Gene set enrichment analysis of TFBS. (a) Gene sets of hallmarks. (b) Gene sets of KEGG.

Using the GSEA method, we observed 6 hallmarks were upregulated in the TFBS-high group, including epithelial-mesenchymal transition, glycolysis, hypoxia, p53 signaling pathway, PI3K/AKT/mTOR signaling pathway, and TNF-alpha signaling via NFKB (Figure 9(a)). Also, we obtained several KEGG terms related to tumor pathways, such as apoptosis, chemokine signaling pathway, and lysosome (Figure 9(b)).

## 4. Discussion

HNSCC is a classical head and neck cancer characterized by extremely heterogeneous features, with a dismal patient outcome [1, 3]. Although increasing evidence unearthed that TRP family genes play a central part in oncogenic effects and cancer therapeutics, an integrated analysis of in-depth expression patterns of TFGs has yet to be clarified. Here, we took advantage of the mRNA expression data of HNSCC to determine significantly prognostic TFGs and create a multibiomarkers signature. Our analyses suggest that the TFGs-based signature could be used for risk stratification and prognosis forecasting in HNSCC, subsequently offering valuable reference for individualized treatment.

Here, we integrated the gene expression profiling of 28 TFGs from the TCGA dataset and built a novel TFBS by Cox hazard regression methods. Survival curves revealed that our proposed TFBS could accurately stratify HNSCC cases into two risk groups with different patient outcomes. Then, ROC curves pointed out the favorable forecasting performance of TFBS. Additionally, the independence of our signature was tested by Cox relative analyses. Furthermore, we successfully generated a nomogram by using the risk score and several clinical factors to expand the predictive ability of TFBS.

In this work, 6 hub TFGs (TRPV4, TRPV2, TRPC1, TRPC6, TRPC3, and TRPM8) were identified to serve as risky factors in HNSCC. TRP channel families are widely expressed in various tissues, regulating the multiple physiological process, and accumulating evidence has pointed out that they contribute to the regulation of carcinogenesis [19, 20]. TRPV4 and TRPV2 both belong to vanilloid receptor-related subfamily. TRPV4 was reported to play crucial roles in maintaining structural integrity of multiple tissues, and its regulatory role in diverse pathological processes in various cancers has been well documented [15, 21–23]. As suggested by Fuji et al., TRPV4 mRNA levels were upregulated in HNSCC cells which showed compromised proliferation capabilities after TRPV4 depletion [24]. It has also been reported that TRPV4 boosts the development of EMT by downregulating E-cadherin expression through AKT and FAK pathways [25]. The prognostic value of TRPV2 has been revealed in certain cancers, including gastric cancer, breast cancer, and endometrial cancer [26–28]. For instance, alteration of the TRPV2 expression level was proven to be a prognostic factor for multiple myeloma cases [29]. Also, TRPV2 overexpression confers a drug-resistant phenotype in gastric cancer, suggesting that promoting tumor cell apoptosis by targeting TRPV2 may be a potential treatment for overcoming drug resistance [30].

Three melastatin-related subfamily genes (TRPC1, TRPC3, and TRPC6) are also recognized as protumoral agents in our signature. Among them, TRPC1 is known for its role in Ca^2+^ influx, cell growth, and migration [31, 32]. In esophageal carcinoma, silencing TRPC1 could repress cell viability and metastasis, indicating that TRPC1 is a protective factor in esophageal cancer [33]. In particular, Osawa et al. once reported that TRPC1 is involved in PI3K activation and could enhance Ca^2+^ concentration, subsequently promoting ERK phosphorylation and cell migration of HSC-3 [34]. Likely, the carcinogenesis of TRPC3 and TRPC6 have been uncovered in several tumors, but their function in HNSCC needs further studies to validate [35–37]. As for melastatin-related subfamily member TRPM8, accumulating evidence has pointed out its crucial role in malignant cells, especially in glioblastoma. TRPM8 was reported to influence the migration capacity of glioblastoma cell by bringing a significant increase in Ca^2+^ concentration, and consistently, TRPM8 downregulation by RNA silencing reduces tumor cell migration capacity and decreases transfilter chemotaxis [38–40].

Our data imply that M2 macrophages and Treg cells are upregulated in the TFBS-high group. In various tumors, intratumoral Treg cell infiltration is observed and is proven to mediate therapeutic resistance in tumor by regulating the activation of Tregs [41–43]. It worth noting that another group of people has pointed out that a unique population of Tregs might exert tissue-specific roles and suppression effects in visceral adipose tissue, distinguishing from their counterparts in lymph nodes [44–46]. Likewise, M2 macrophages play a central part in tumor progression and metastasis [47, 48]. Regarding HNSCC, Saloura et al. have once tested Treg markers and M2 macrophages markers expression in HNSCC specimens and normal controls and found that these targets were notably upregulated in HNSCC [49]. Similar results were observed in another study, indicating that M2 and Treg infiltration affect the HNSCC development [50]. Overall, the Treg cells activities and M2 macrophages polarization are likely to directly impact the therapeutic outcome, and future studies need to focus on establishing the precise effect of immunocyte activation in HNSCC carcinogenesis.

GSEA was implemented to detect of the function of differential gene sets in HNSCC. The epithelial-mesenchymal transition, glycolysis, hypoxia, and TNF-alpha signaling via NFKB gene sets are recognized as the most positively enriched in the HNSCC dataset. Epithelial-mesenchymal transition pathway mainly involves the expression change of cadherin relevant proteins. In the EMT process, enhanced mesenchymal phenotype as well as decreased epithelial phenotype together leads to functional changes in tumor cell migration and invasion [51]. Chen et al. uncovered that higher expression of CMTM6 revealed a dismal outcome of HNSCC patients, and silencing CMTM6 could inhibit EMT and tumor stemness, suggesting it might be a favorable biomarker for HNSCC management [52]. SOX8, a member of the SOX family, displayed a higher expression level in chemoresistance HNSCC cells. As revealed by Xie et al., inhibiting SOX8 could enhance cell sensitivity to cisplatin and repressed EMT by targeting the Wnt/*β*-catenin pathway [53]. In HNSCC, enhanced glycolysis as evidenced by more serum pyruvic acid production was observed in patients with increasing clinical stage and advancing histopathological grades [54]. For instance, SKA3 was proved to be bound up with advanced stage in laryngeal cancer and determined as a novel marker which had a carcinogenic effect. Gao et al. observed that SKA3 knockdown could weaken cell growth and chemoresistance in a PLK1-induced glycolysis way [55]. In addition, PER1 was reported to get involved in glycolysis and glucose uptake in oral carcinoma, which in turn regulate cell viability by targeting RACK1-based complex [56]. Hypoxia has been studied to be responsible for tumor progression and drug resistance in HNSCC, and another HNSCC study mentioned that the hypoxia inducible transcription factor was positively associated with tumor growth [57, 58]. The PI3K/AKT/mTOR pathway has emerged as one of the most frequently altered in human cancer [59, 60]. For example, Sanjukta and his colleagues confirmed the involvement of mTOR signaling related genes in the pathogenesis of HNSCC. PI3K inhibitors displayed dose-dependent suppression of cell viability in HNSCC [61, 62]. To sum up, our results indicate the enrichment of above pathways in HNSCC, which may open novel therapeutic options in future treatment.

Inevitably, in our project remains several shortcomings. First, we endeavor to collect additional HNSCC queues to confirm the reliability of our TFBS. Unfortunately, there is no available dataset for lncRNA-based model verification. Second, more clinical potency needs to be further developed. Therefore, we will uncover the response of chemotherapy between both risk groups and forecast the possible small molecular drugs for HNSCC cases based on our established TFBS. In addition, some new immunotherapy relevant databases are warranted to validate our results in future studies.

Third, in our project, the expression pattern of our model will be estimated by various wet experiments, such as immunohistochemistry. Moreover, we will detect the molecular mechanism of the TFBS using in vitro analysis.

In conclusion, we successfully created a TRP family gene-based signature for HNSCC patients. Our established TFBS might offer a precise and powerful prediction option for the patient outcome of HNSCC. The biomarkers determined in TFBS could mirror the immune landscape of cases, which may provide immune therapeutic strategy for HNSCC.

## Figures and Tables

**Table 1:** Six TFGs-based signature markedly correlated with patient outcome.

Gene	Coefficient	Hazard ratio (95% CI)	*P* value
TRPC1	0.2075	1.27 (1.04–1.53)	0.015
TRPC3	0.1439	1.42 (1.04–1.94)	0.026
TRPC6	0.1553	1.32 (1.14–1.54)	<0.001
TRPM8	0.2698	1.27 (1.10–1.48)	0.001
TRPV2	0.2947	1.97 (1.26–3.08)	0.003
TRPV4	0.0778	1.62 (1.16–2.28)	0.004

## Data Availability

The public datasets to support the results of this subject are available from TCGA (https://portal.gdc.cancer.gov/) and STRING (https://cn.string-db.org/).

## Supplementary Materials

Supplementary Materials Supplementary Table 1: the list of 28 TRP family genes.
