# Supplementary material for: A TRP Family Based Signature for Prognosis Prediction in Head and Neck Squamous Cell Carcinoma
Source: J Oncol. 2022 Jan 31;2022:8757656. doi: 10.1155/2022/8757656 (PMC8820906; doi:10.1155/2022/8757656)
Supplement: Supplementary Materials — Supplementary Table 1: the list of 28 TRP family genes. [file 8757656.f1.pdf]

Supplementary Table S1 The list of 28 TRP family genes.

Gene Name	Symbol ID
TRPM1	TRPM1
TRPM2	TRPM2
TRPM3	TRPM3
TRPM4	TRPM4
TRPM5	TRPM5
TRPM6	TRPM6
TRPM7	TRPM7
TRPM8	TRPM8
TRPC1	TRPC1
TRPC2	TRPC2
TRPC3	TRPC3
TRPC4	TRPC4
TRPC5	TRPC5
TRPC6	TRPC6
TRPC7	TRPC7
TRPV1	TRPV1
TRPV2	TRPV2
TRPV3	TRPV3
TRPV4	TRPV4
TRPV5	TRPV5
TRPV6	TRPV6
TRPA1	TRPA1
TRPP2	PKD2
TRPP3	PKD2L1
TRPP5	PKD2L2
TRPML1	MCOLN1
TRPML2	MCOLN2
TRPML3	MCOLN3
